# Efficacy of Chinese Herbal Medicine for Diarrhea-Predominant Irritable Bowel Syndrome: A Meta-Analysis of Randomized, Double-Blind, Placebo-Controlled Trials

**DOI:** 10.1155/2016/4071260

**Published:** 2016-07-31

**Authors:** Jia-Jie Zhu, Shan Liu, Xiao-Lan Su, Zi-Song Wang, Yu Guo, Yi-Jie Li, Yang Yang, Li-Wei Hou, Qing-Guo Wang, Ru-Han Wei, Jian-Qin Yang, Wei Wei

**Affiliations:** ^1^Beijing Key Laboratory of Functional Gastrointestinal Disorders Diagnosis and Treatment of Traditional Chinese Medicine, Wangjing Hospital, China Academy of Chinese Medical Sciences, Beijing 100102, China; ^2^Dongzhimen Hospital, Beijing University of Chinese Medicine, Beijing 100700, China; ^3^Basic Medical College, Beijing University of Chinese Medicine, Beijing 100029, China; ^4^Cleveland State University, Cleveland, OH 44115-2214, USA

## Abstract

*Objective*. To explore the efficacy of Chinese herbal medicine in treating diarrhea-predominant irritable bowel syndrome (D-IBS).* Methods*. Four English and four Chinese databases were searched through November, 2015. Randomized, double-blind and placebo-controlled trials were selected. Data extraction and quality evaluation were performed by two authors independently. RevMan 5.2.0 software was applied to analyze the data of included trials.* Results*. A total of 14 trials involving 1551 patients were included. Meta-analysis demonstrated superior global symptom improvement (RR = 1.62; 95% CI 1.31, 2.00; *P* < 0.00001; number needed to treat = 3.6), abdominal pain improvement (RR = 1.95; 95% CI 1.61, 2.35; *P* < 0.00001), diarrhea improvement (RR = 1.87; 95% CI 1.60, 2.20; *P* < 0.00001), pain threshold assessment (MD = 54.53; 95% CI 38.76, 70.30; *P* < 0.00001), and lower IBS Symptom Severity Score (SMD = −1.01; 95% CI −1.72, −0.30; *P* = 0.005), when compared with placebo, while for defecation threshold assessment, quality of life, and adverse events, no differences were found between treatment groups and controlled groups.* Conclusion*. This meta-analysis shows that Chinese herbal medicine is an effective and safe treatment for D-IBS. However, due to the small sample size and high heterogeneity, further studies are required.

## 1. Introduction

Irritable bowel syndrome (IBS), the most common functional gastrointestinal disorder across the world, is characterized by recurrent abdominal pain or discomfort associated with disturbances in defecation and could not be explained by any structural or anatomical abnormality [1, 2]. According to the different bowel behaviors, IBS could be divided into four subtypes, namely IBS-C (constipation-predominant), IBS-D (diarrhea-predominant), IBS-M (mixed), and IBS-U (unspecified) [2], among which IBS-D is the major subtype [3]. With the high prevalence of 14%~28% among Europe [4] and 0.82%~11.5% in China [5, 6], it has serious influences on the quality of life of patients and costs a large amount of medical resources (1007.3 million in 2004), which is close to 25% of the total cost of all functional GI disorders (3988.8 million) [7].

Although with a great progress in the understanding of IBS [8], conventional treatments, including antidiarrheals, antispasmodics, antidepressants, probiotics and psychological treatments [9–12], were still limited in clinic because of side effects, costly medication expenses, and high relapse rates [13] and seemed to be unsuccessful to improve the quality of IBS patients' life [14].

Hence, an increasing number of patients (from 16% in 1986 to 51% in 2005) tend to use complementary and alternative medicine (CAM) [15]. Chinese herbal medicine (CHM), the major part of CAM and characterized by syndrome differentiation and treatment, has widely been accepted during last few decades [16]. Several clinical trials have been conducted, but the results were inconsistent [15, 17–19]. Although several systematic reviews have shown a therapeutic benefit, the efficacy of CHM was still controversial due to the poor qualities of the original studies, and these authors also emphasized that it was premature to recommend herbal medicines for routine use in IBS [20, 21].

Recently, a high quality meta-analysis, which focused on soothing the liver and invigorating the spleen therapy, has demonstrated CHM is an effective treatment for IBS-D [22]. According to a literature review, spleen-stomach weakness (57.5%),* yang* deficiency of the spleen and kidney (52.5%), stagnation of liver* qi*, and deficiency of the spleen (52.5%) are the most common Traditional Chinese Medicine (TCM) syndromes in IBS-D [23]. In other words, soothing the liver, invigorating the spleen, and warming the kidney are the main therapies for IBS-D. Given all the information, a meta-analysis of randomized, double-blind, placebo-controlled trials is required to confirm whether CHM is beneficial to IBS-D patients.

## 2. Methods

The registered protocol of this systematic review could be found in the PROSPERO database (http://www.crd.york.ac.uk/PROSPERO/display_record.asp?ID=CRD42015029540).

### 2.1. Search Strategy

Two researchers searched four English electronic databases and four Chinese electronic databases from their establishments through November 2015, including PubMed, Web of Science, Cochrane Library, Embase, Chinese Biomedicine (CBM), China National Knowledge Infrastructure (CNKI), Chinese Scientific Journals Database (VIP), and WanFang Database. Conference proceedings and dissertations which involved unpublished trials were also searched from CNKI and WanFang databases.

The following search terms, or the Chinese equivalent for Chinese databases, were used singly and combinedly depending on which database was searched: “Irritable Bowel Syndrome”, “Irritable Bowel Syndromes”, “Syndrome, Irritable Bowel”, “Syndromes, Irritable Bowel”, “Traditional Chinese Medicine”, “Medicine, Chinese Traditional”, “Chinese Traditional Medicine”, “Chinese Medicine, Traditional”, “TCM”, “Herbal Medicine”, “Medicine, Herbal”, “herb^*∗*^”, “randomized”, “placebo”, “double-blind” and “double-blinded”.

 #1 Search (((((Irritable Bowel Syndrome [MeSH Terms]) OR Irritable Bowel Syndrome [Title/Abstract]) OR Irritable Bowel Syndromes [Title/Abstract]) OR “Syndrome, Irritable Bowel” [Title/Abstract]) OR “Syndromes, Irritable Bowel” [Title/Abstract]).
 #2 Search ((((((((((Traditional Chinese Medicine [Title/Abstract]) OR “Chinese Medicine, Traditional” [Title/Abstract]) OR “Medicine, Chinese Traditional” [Title/Abstract]) OR Chinese Traditional Medicine [Title/Abstract]) OR Herbal Medicine [MeSH Terms]) OR Herbal Medicine [Title/Abstract]) OR “Medicine, Herbal” [Title/Abstract]) OR TCM [Title/Abstract]) OR herb^*∗*^ [Title/Abstract]) OR “Medicine, Chinese Traditional” [Mesh]).
 #3 Search ((randomized [Title/Abstract]) AND placebo [Title/Abstract]) AND ((double-blind [Title/Abstract]) OR double-blinded [Title/Abstract]).
 #1 and #2 and #3.

## 3. Inclusion/Exclusion Criteria

### 3.1. Types of Studies

Studies, performed as randomized, double-blind, placebo-controlled trials, which compared the efficacy and safety of CHM with placebo for IBS-D were included. English and Chinese were applied as language restriction.

### 3.2. Types of Participants

Patients were diagnosed with IBS-D according to the ROME I, II, or III criteria.

### 3.3. Types of Interventions

Orally administered CHM, in any preparations like capsules, decoctions, extracted granules, and oral liquids, were used alone in the treatment groups. The controlled groups only received placebos which were similar to the herbal medicines in taste, smell, and look. Treatment durations were not limited.

### 3.4. Types of Outcome Measures

Primary outcomes were global syndrome improvement, IBS Symptom Severity Score (SSS). Secondary outcomes were abdominal pain improvement, diarrhea improvement, visceral hypersensitivity assessment, quality of life, and adverse events.

## 4. Study Selection and Data Extraction

According to the inclusion and exclusion criteria, study selection and data extraction were carried out by two researchers independently. The detailed information including diagnostic criteria for IBS-D, TCM syndrome, TCM therapy, population, baseline characteristics, details of the interventions, followup time, and outcome measurements were extracted to form a conclusive table. Any divergences were resolved by discussion and consensus with a third researcher.

### 4.1. Assessment of Risk Bias

Using the Cochrane risk of bias tool, the methodological qualities of included trials were evaluated by two researchers, respectively [24]. The judgment of the other bias includes comparable baseline characteristic, for-profit, and inclusion and exclusion criteria into consideration. Disagreements were resolved through discussion and consensus with a third researcher.

### 4.2. Data Analysis

RevMan 5.3 was the utilized software to analyze the data. We took dichotomous data as Relative Risk (RR) and continuous variables as Mean Difference (MD) with 95% Confidence Intervals (CI). Standardized Mean Difference (SMD) analyses were performed when different measurement scales were used. Only the first phase outcome data were analyzed in cross-over trials. Both the Chi-squared (*χ* ^2^) test and *I*-squared (*I* ^2^) statistic were used for the assessment of heterogeneity [25]. If a significant heterogeneity existed (*I* ^2^ > 50% or *P* < 0.05), a random effect model was performed to calculate the pooled RR. Otherwise, a fixed effect model was used [26]. In order to inquire into the origin of heterogeneity among studies, a sensitivity analysis was conducted by omitting one trial successively. The Number Needed to Treat (NNT) was calculated as the reciprocal of the therapeutic gain. Subgroup analysis for different TCM therapies was performed when the necessary data were available.

## 5. Result

### 5.1. Study and Selection

**Figure 1:**
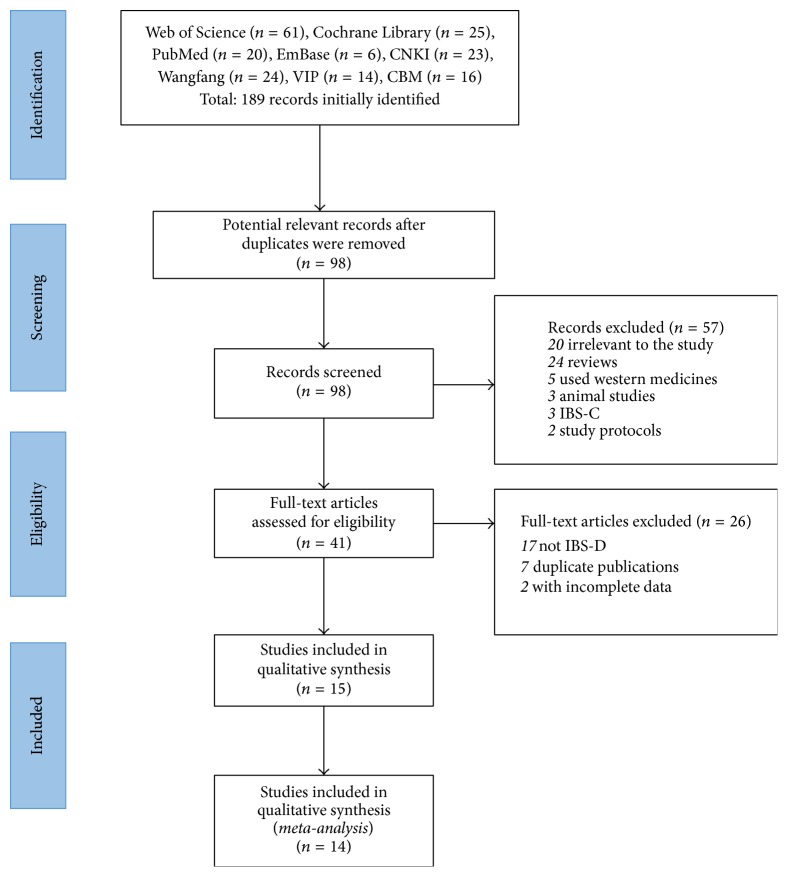
Flow chart and study selection.

A total of 196 citations were identified for initial search and 15 articles, in which 2 articles [29, 30] reported 1 trial, were involved at last (Figure 1).

### 5.2. Description of Study

The 15 articles, 12 journal papers, 3 dissertations, and 1 conference proceeding contained 1551 subjects (922 in trail group and 629 in control group). Among them 4 studies were conducted in Australia [27], Korean [39], Hong Kong [18] and Chinese Mainland [37], respectively, and published in English. The remaining [28–36, 38, 40] were all completed in China and published in Chinese. All of the trials had 2 arms, except 1 trial [27] that had 3 arms, standard, individualized, and placebo group. Three TCM therapies, soothing the liver and invigorating the spleen (SLIS), warming the kidney and invigorating the spleen (WKIS), and individualized therapies were involved.  Table 1 showed the detailed information of the included trials. The ingredients of herbal formulae were listed in Table 2.

### 5.3. Methodological Quality

**Figure 2:**
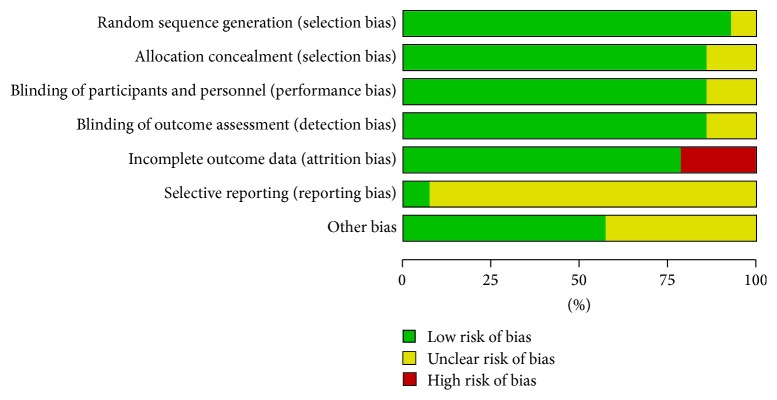
Methodological quality assessment of the risk of bias for each included study.

All included studies were designed as randomized studies. Ten studies [18, 27, 28, 31, 33–35, 38–40] used random number tables or lists, 1 study [29, 30] used draw by lots, while the remaining 3 did not mention the specific methods. We tried to contact the authors and they confirmed that 2 studies [36, 37] used random number tables, but the others [32] did not respond. All the studies used sealed envelopes except 2 [29, 32] which had no details about the blending method and did not respond to our emails. 11 trials [18, 27, 31, 33–40] reported drop-out of patients, but Intention-to-Treat (ITT) analyses were not performed in 4 trials [27, 36–38], in which we just managed to complete it for 1 trial [37]. Although all trials reported all the outcome measurements mentioned in the methods, we evaluated them as unclear risk due to the inaccessibility of the protocols except 1 trial [39]. For other bias, 6 studies [18, 27, 31, 34, 39, 40] were rated as unclear risk because of the lack of ages and disease durations (Figure 2).

### 5.4. Global Symptom Improvement

**Figure 3:**
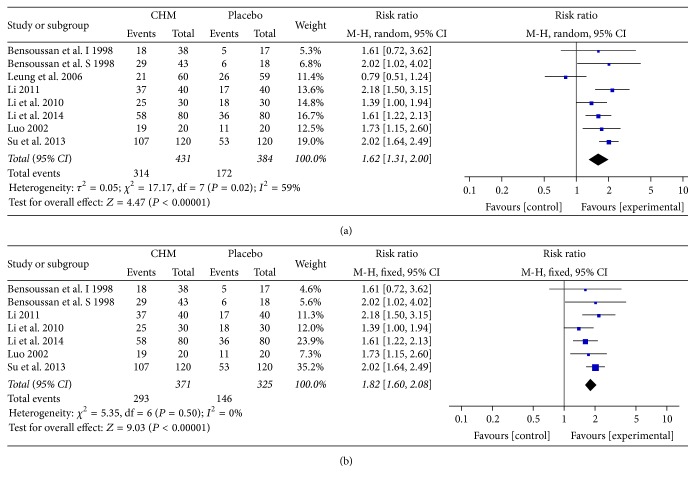
(a) Forest plot of global symptom improvement in patients with IBS-D treated with CHM compared to placebo. (b) Sensitivity analysis was performed by omitting one study.

Seven trials [18, 27, 28, 33, 36, 37, 40] reported the global symptom improvement and 1 trial [27] was counted as two comparisons because it had three arms. A total of 815 patients (431 in CHM groups and 384 in placebo groups) were included in the analysis. With a statistical significance, the result demonstrated that CHM had a superior efficacy in global symptom improvement than placebo (RR = 1.62; 95% CI 1.31, 2.00; *P* < 0.00001) (Figure 3(a)). A 28.1% of therapeutic gain was exhibited between the comparison of CHM and placebo (72.9% versus 44.8%; NNT = 3.6) (Table 3). A sensitivity test was conducted due to the high heterogeneity (*I* ^2^ = 59%, *P* = 0.02). It showed that Leung et al.'s [18] study may be the major origin of the heterogeneity. After omitting Leung et al.'s study, the result still supported the previous consequence (RR = 1.82; 95% CI 1.60, 2.08; *P* < 0.00001) with a low heterogeneity (*I* ^2^ = 0%, *P* = 0.50) (Figure 3(b)).

**Figure 4:**
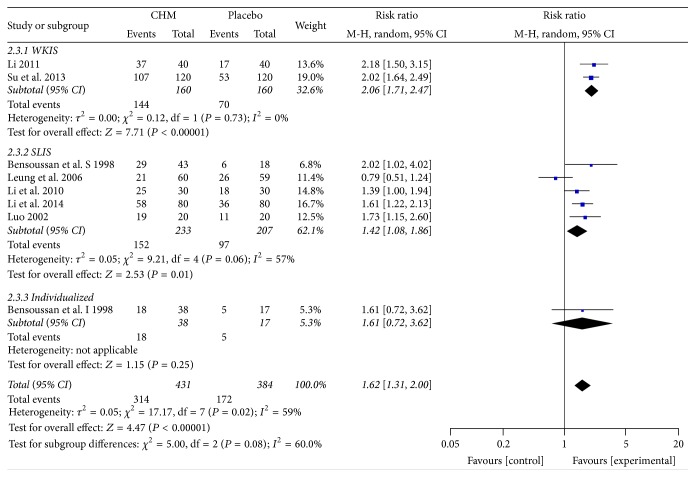
Forest plot of global symptom improvement in patients with IBS-D treated with CHM compared to placebo, subgroup analysis.

In addition, a subgroup analysis was implemented according to the different therapies. The result showed that WKIS therapy (RR = 2.06; 95% CI 1.71, 2.47; *P* < 0.00001) and SLIS therapy (RR = 1.42; 95% CI 1.08, 1.86; *P* = 0.01) both were effective compared with placebo (Figure 4). But the heterogeneity was still significant in SLIS group (*I* ^2^ = 57%, *P* = 0.06), originating from Leung et al.'s [18] study again.

### 5.5. IBS-SSS

**Figure 5:**
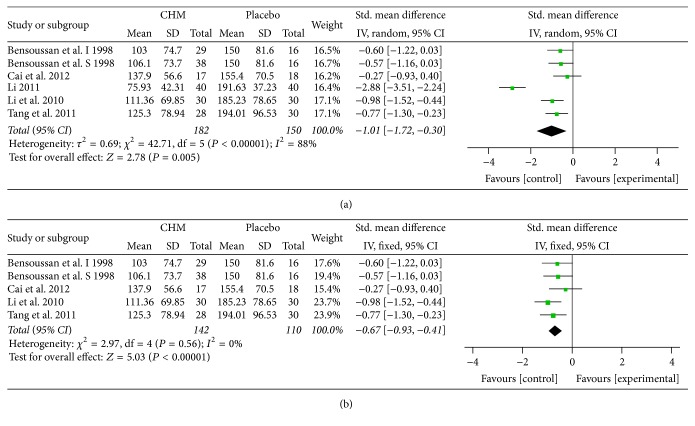
(a) Forest plot of IBS-SSS improvement in patients with IBS-D treated with CHM compared to placebo. (b) Sensitivity analysis was performed by omitting one study.

5 studies [27, 33, 35, 36, 38] including 6 comparisons reported the IBS-SSS. The reduction of the SSS showed that the severity of IBS symptoms was substantially relieved by CHM compared to placebo (SMD = −1.01; 95% CI −1.72, −0.30; *P* = 0.005) (Figure 5(a)). The heterogeneity was high (*I* ^2^ = 88%, *P* < 0.00001). Sensitivity test indicated that Li's [36] study may be the main contribution. After exclusion of Li's study the heterogeneity decreased straightly (*I* ^2^ = 0%, *P* = 0.56) while the result was not obviously altered (SMD = −0.67; 95% CI −0.93, −0.41; *P* < 0.00001) (Figure 5(b)).

### 5.6. Abdominal Pain Improvement

**Figure 6:**
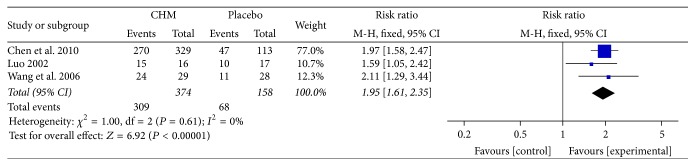
Forest plot of abdominal pain improvement in patients with IBS-D treated with CHM compared to placebo.

Three studies [28, 31, 34] reported the abdominal pain improvement. With a significant difference, CHM had a better abdominal pain improvement than placebo (RR = 1.95; 95% CI 1.61, 2.35; *P* < 0.00001) and no observed heterogeneity existed (*I* ^2^ = 0%, *P* = 0.61) (Figure 6).

### 5.7. Diarrhea Improvement

**Figure 7:**
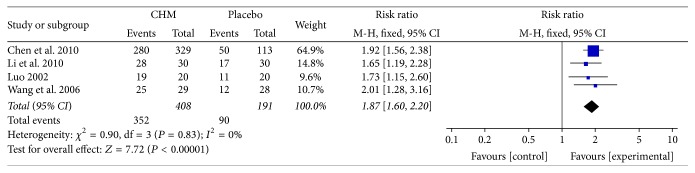
Forest plot of diarrhea improvement in patients with IBS-D treated with CHM compared to placebo.

Four studies [28, 31, 33, 34] reported diarrhea improvement. CHM showed conspicuous improvement for diarrhea compared with placebo (RR = 1.87; 95% CI 1.60, 2.20; *P* < 0.00001). No obvious heterogeneity was seen (*I* ^2^=0%, *P* = 0.83) (Figure 7).

### 5.8. Visceral Hypersensitivity Assessment (Pain Threshold and Defecation Threshold)

**Figure 8:**
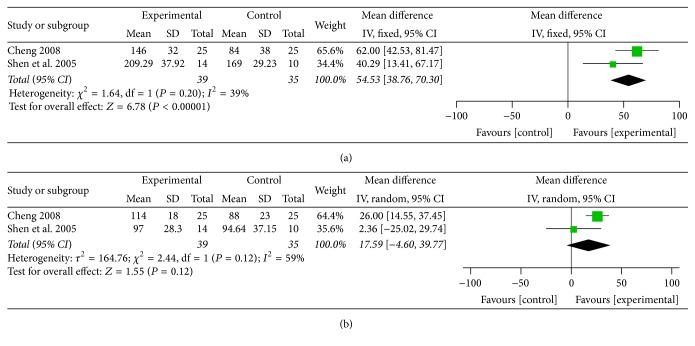
(a) Forest plot of pain threshold improvement in patients with IBS-D treated with CHM compared to placebo. (b) Forest plot of defecation threshold improvement in patients with IBS-D treated with CHM compared to placebo.

Two studies [30, 32] used anorectal manometry to evaluate the visceral hypersensitivity of patients. CHM showed a superior improvement in pain threshold than placebo (MD = 54.53; 95% CI 38.76, 70.30; *P* < 0.00001) with a moderate heterogeneity (*I* ^2^ = 39%, *P* = 0.20) (Figure 8(a)). As for defecation threshold, the result did not show a significant improvement than placebo (MD = 17.59; 95% CI −4.60, 39.77; *P* = 0.12). And the heterogeneity was high (*I* ^2^ = 59%, *P* = 0.12) (Figure 8(b)).

### 5.9. IBS-QOL Score

**Figure 9:**
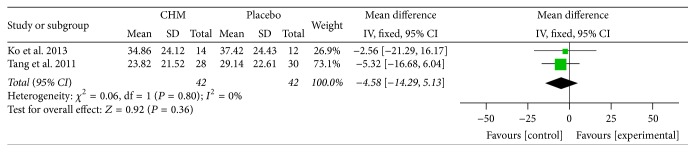
Forest plot of IBS-QOL score in patients with IBS-D treated with CHM compared to placebo.

Three studies assessed the quality of life of the patients. Two [35, 39] used the IBS Quality of Life Questionnaire (IBS-QOL), while the other [18] used the validated Hong Kong Chinese version of the Short Form 36 (SF-36). Because of the different instruments, we pooled two studies. No advantage has been found in CHM compared with placebo (MD = −4.58; 95% CI −14.29, 5.13; *P* = 0.36). No observed heterogeneity was seen (*I* ^2^ = 0%, *P* = 0.80) (Figure 9). In addition, Leung et al.'s [18] study also showed no remarkable difference in the health-related life between CHM and placebo group.

### 5.10. Adverse Events

Ten studies mentioned the adverse events and 5 [33, 35–38] reported no adverse event occurred. Bensoussan et al. [27] reported 2 patients withdrew due to upper gastrointestinal discomfort and headaches, respectively, in standard treatment group. Wang et al. [31] reported 1 flush and abdominal pain case. Leung et al. [18] reported 2 patients had skin rash and thyroiditis in TCM group and 1 had facial nerve palsy in placebo group. In Chen et al.'s study [34], 2 mild nausea and mild pruritus cases were noted. And 2 cases of headache, 1 case of low-back pain, and 1 case of dysmenorrhea were reported by Ko et al. [39]. No difference of adverse events was observed between CHM and placebo.

## 6. Discussion 

### 6.1. Main Findings

This meta-analysis investigated the efficacy of CHM in the treatment of IBS in comparison to placebo. The results demonstrated that CHM had superior improvements in global symptom (RR = 1.62), IBS-SSS (SMD = −1.01), diarrhea (RR = 1.87), abdominal pain (RR = 1.95), and pain threshold (MD = 54.53), with no superiority in quality of life, defecation threshold, and a seldom adverse events occurrence.

In subgroup analysis SLIS, WKIS and individualized groups' therapeutic gains over placebo were 18.3%, 46.2%, and 18.8%, and the NNT were 5.5, 2.2, and 5.6, respectively. That being said, WKIS seemed to be the best therapy for IBS-D. But as we all know, syndrome differentiation and treatment are the core of TCM. The efficacy of TCM derives from the accuracy of syndrome differentiation [23]. In Bensoussan et al.'s [27] study, no significant difference was noticed between the standard group and individualized group at the end of the 8-week procedure. But the individualized group maintained a better improvement after a 14-week followup. Therefore, using TCM syndrome differentiation is still required to enhance the pertinence of treatment.

Anorectal manometry was used to assess the visceral hypersensitivity. CHM could significantly increase the pain threshold. That meant CHM could reduce visceral pain. While meta-analysis did not show an advantage in defecation threshold between CHM and placebo, both of the two studies showed that the CHM groups had significant improvements while placebo groups had not. In Shen et al.'s [30] study, the initial defecation threshold in CHM group (79.29 ± 34.11 mL) was lower than the placebo group (87.00 ± 21.00 mL). Although CHM significantly improved the threshold (97.00 ± 28.30 mL) after the treatment, it was approximated to the placebo group (94.64 ± 37.15 mL). However, due to the small samples, it is difficult to determine a conclusion on this issue.

Substantial heterogeneity was found in global symptom improvement and IBS-SSS. The sensitive tests indicated that Leung et al.'s [18] study and Li's [36] study were the main causes separately. After checking all the studies carefully, three differences were found between Leung et al.'s study and the others'. First of all, in Leung et al.'s formula, two heat-clearing herbs,* Portulaca oleracea* (Ma Chi Xian) and* Coptis chinensis* (Huang Lian), were added in. These herbs were not suitable for the syndrome of liver* qi* stagnation and spleen deficiency and could lead to diarrhea. In addition, Leung et al.'s study has the lowest response rate (35.0% in CHM group; 44.1% in placebo group) and the highest withdrawal rate (23.3% in CHM group; 16.9% in placebo group) compared with the others. These might result from the inappropriate formula and could account for the heterogeneity.

In Li's study, the disease durations were shorter than the other four studies [29, 33, 35, 38]. This may contribute to the heterogeneity mostly. In addition, the different TCM syndromes and therapies also could be a matter of heterogeneity.

### 6.2. Interpretation

With the deepening of the research, an increasing number of mechanisms of CHM in treating IBS-D were revealed. The effective targets included the regulation of hormones and cytokines in the enteric nervous system, the adjustment of the brain-gut axis, and the modulation of the gut motility [41]. Besides, in Ko et al.'s [39] study, Huo Xiang Zheng Qi San (a CHM formula) showed a tendency to have a lower Firmicutes/Bacteroidetes ratio and intestinal permeability index, which could relieve the IBS symptoms. Increased expressions of CD45+ and CD3+ and a decreased CD4+/CD8+ ratio, meaning an immunity disorder, were found in IBS rats, while CHM, which acted to warming the kidney and invigorating the spleen, could reduce the expressions of CD45+ and CD3+ and increase the CD4+/CD8+ ratio, indicating a regulative effect in immune response [42].

Cheng [32] and Shen et al. [30] studies both showed an improvement in visceral hypersensitivity, which was caused by a variety of factors and was believed to have a large contribution to the genesis of IBS [43]. This result may through the reduction of serotonin (5-HT) both in serum and enteric mucosa [32] lead to a relief of visceral pain [30].

### 6.3. Strengths and Limitations

Several strengths were contained in this meta-analysis. First, this is a systematic review on a significant issue of human health. Second, the inclusion and exclusion criteria were strict and the methodological quality of the included trials was commonly rated as high after a rigorously assessment. Furthermore, a standard protocol of this meta-analysis was registered and published in PROSPERO database. However, this meta-analysis still had some limitations. First, because of the strict inclusion criteria, the suitable trials were few and the sample sizes were small. Second, 12 out of 14 trials were carried out in China and 10 studies were printed in Chinese. A funnel plot analysis was not performed successfully due to inadequate number of included studies in meta-analysis, so potential publication bias may exist. Third, owing to insufficient suitable literatures, this meta-analysis did not involve other TCM syndromes such as cold-heat in complexity and spleen-stomach weakness. Fourth, the course of treatment, ranging from 3 to 16 weeks, as well as the follow-up duration, from 2 to 14 weeks, was not long enough to appraise the efficacy and safety of CHM.

### 6.4. Implications for Further Study

Although all the studies were generally well designed, several issues still should be addressed to improve the methodological quality of the clinical studies. First, a sample size calculation should be performed before enrollment. Second, randomization, allocation concealment, and blinding methods should be described expressly and reported fully in the article. Third, withdrawal/dropout during the study and use of ITT analysis should be reported clearly. Fourth, due to the relapsed nature of IBS, a sufficient followup duration is required to evaluate the long-term efficacy. Fifth, a link of a registered protocol is required in the article.

## 7. Conclusion

From the above, this meta-analysis demonstrates that SLIS and WKIS are feasible, effective, and safe treatments superior to placebo in improving global symptoms, IBS-SSS, abdominal pain, diarrhea, and visceral hypersensitivity with IBS-D. However, due to the small sample size and the high heterogeneity, a confirmative conclusion is still premature. In future studies, larger sample sizes and longer courses should be undertaken to perfect the studies.

## Figures and Tables

**Table 1:** The characteristics of the included studies.

Author (year)	Diagnosis criteria	TCM syndrome	*N*	Age (year)		Disease duration (months)		TCM therapy	Outcome measurements	Duration (weeks)	Followup (weeks)
			(T versus C)	T	C	T	C				
Bensoussan et al. (1998) [27]	Rome I	Stagnation of liver *qi* and deficiency in the spleen	43 versus 17	47.60 ± 15.10	45.0 ± 13.9	NR	NR	Soothing the liver and invigorating the spleen	(1) BSS score; (2) global symptom improvement	16 w	14 w
		NR	38 versus 18	47.40 ± 13.40		NR	NR	Individualized			

Luo (2002) [28]	Rome II	Stagnation of liver *qi* and deficiency in the spleen	20 versus 20	36.90 ± 15.10	37.80 ± 13.40	35.50 ± 18.90	34.60 ± 20.20	Soothing the liver and invigorating the spleen	(1) Global symptom improvement; (2) serum and mucosal VIP	4 w	NR

Shen et al. (2005) [29, 30]	Rome II	Stagnation of liver *qi* and deficiency in the spleen	14 versus 10	55.50 ± 28.60	51.90 ± 13.80	25.50 ± 15.70	26.80 ± 15.30	Soothing the liver and invigorating the spleen	(1) Anorectal manometry; (2) functional MR test; (3) global symptom score	4 w	NR

Wang et al. (2006) [31]	Rome II	Stagnation of liver *qi* and deficiency in the spleen	29 versus 28	37.10 ± 10.40	36.90 ± 8.90	NR	NR	Soothing the liver and invigorating the spleen	(1) Abdominal pain improvement; (2) diarrhea improvement; (3) TCM syndrome improvement	3 w	4 w

Leung et al. (2006) [18]	Rome II	Stagnation of liver *qi* and deficiency in the spleen	60 versus 59	45.40 ± 11.90	43.60 ± 13.90	NR	NR	Soothing the liver and invigorating the spleen	(1) Global symptom improvement; (2) individual symptom score; (3) daily bowel frequency; (4) SF-36	8 w	8 w

Cheng (2008) [32]	Rome II	Stagnation of liver *qi* and deficiency in the spleen	25 versus 25	36.32 ± 12.17	33.68 ± 10.81	40.56 ± 26.04	38.04 ± 28.32	Soothing the liver and invigorating the spleen	(1) Anorectal manometry; (2) serum and mucosal 5-HT	4 w	NR

Li et al. (2010) [33]	Rome III	Stagnation of liver *qi* and deficiency in the spleen	30 versus 30	40.57 ± 14.06	36.67 ± 14.49	97.68 ± 14.28	99.00 ± 14.52	Soothing the liver and invigorating the spleen	(1) IBS-SSS; (2) global IBS symptom improvement	4 w	4 w

Chen et al. (2010) [34]	Rome III	Stagnation of liver *qi* and deficiency in the spleen	360 versus 120	NR	NR	NR	NR	Soothing the liver and invigorating the spleen	(1) Diarrhea improvement; (2) abdominal pain improvement	3 w	NR

Tang et al. (2011) [35]	Rome III	NR	30 versus 30	47.68 ± 12.98	46.13 ± 13.01	79.75 ± 103.64	107.60 ± 94.96	Soothing the liver and invigorating the spleen	(1) IBS-SSS; (2) IBS-QOL	8 w	NR

Li (2011) [36]	Rome III	*Yang* deficiency of the spleen and kidney	41 versus 41	40.95 ± 11.42	39.98 ± 11.45	37.95 ± 14.55	37.70 ± 15.13	Warming the kidney and invigorating the spleen	(1) Global symptom improvement; (2) IBS-SSS	4 w	1 m

Su et al. (2013) [37]	Rome III	*Yang* deficiency of the spleen and kidney	120 versus 120	38.00 ± 12.00	37.00 ± 12.00	38.00 ± 15.00	36.00 ± 17.00	Warming the kidney and invigorating the spleen	(1) Global symptom improvement; (2) TCM symptom improvement; (3) recurrence rate	4 w	6 m

Cai et al. (2012) [38]	Rome III	Stagnation of liver *qi* and deficiency in the spleen	18 versus 19	43.24 ± 10.26	41.89 ± 9.33	54.72 ± 53.04	59.76 ± 60.12	Soothing the liver and invigorating the spleen	(1) IBS-SSS; (2) TCM syndrome score	8 w	NR

Ko et al. (2013) [39]	Rome III	NR	14 versus 12	47.50 ± 13.60	47.50 ± 16.00	NR	NR	Resolving dampness to move qi	(1) Adequate relief (AR); (2) proportion of responders (PR); (3) IBS-QoL; (4) patient diary	8 w	2 w

Li et al. (2014) [40]	Rome III	NR	80 versus 80	NR	NR	NR	NR	Soothing the liver and invigorating the spleen	Global symptom improvement	4 w	NR

TCM, Chinese Traditional Medicine; T, trial group; C, control group; NR, no report.

**Table 2:** The ingredients of each formula.

Studies	Ingredients of each formula					
Bensoussan et al. (1998) [27]	Standard formula	*Codonopsis pilosulae* (Dang Shen)	*Agastaches seu Pogostemi* (Huo Xiang)	*Ledebouriellae sesloidis* (Fang Feng)	*Coicis lachryma-jobi* (Yi Yi Ren)	Bupleurum chinense (Chai Hu)
		*Artemesiae capillaris *(Yin Chen)	*Atractylodis macrocephalae *(Bai Zhu)	*Magnoliae officinalis *(Hou Po)	*Citri reticulatae *(Chen Pi)	*Zingiberis officinalis *(Pao Jiang)
		*Fraxini* (Qin Pi)	*Poriae cocos *(Fu Ling)	*Angelica dahurica *(Bai Zhi)	*Plantaginis *(Che Qian Zi)	*Phellodendri* (Huang Bai)
		*Glycyrrhizae uralensis* (Zhi Gan Cao)	*Paeoniae lactiflorae* (Bai Shao)	*Saussureae seu vladimirae* (Mu Xiang)	*Coptidis *(Huang Lian)	*Schisandrae *(Wu Wei Zi)
	Individual group	81 individual dried powdered Chinese herbs				

Luo (2002) [28]		*Bupleunrum chinensie *(Chai Hu)	*Fructus aurantii *(Zhi Qiao)	*Paeoniae alba *(Bai Shao)	*Saposhnikovia divaricata *(Fang Feng)	*Radix Codonopsis* (Dang Shen)
		*Fructus mume *(Wu Mei)	*Citri reticulatae* (Chen Pi)	*Saussureae seu vladimirae* (Mu Xiang)	*Atractylodes macrocephala* (Bai Zhu)	*Glycyrrhizae uralensis* (Zhi Gan Cao)

Shen et al. (2005) [29, 30]		*Atractylodes macrocephala* (Bai Zhu)	*Paeoniae alba *(Bai Shao)	*Saposhnikovia divaricata *(Fang Feng)	*Fructus mume *(Wu Mei)	*Glycyrrhizae preparata *(Zhi Gan Cao)

Wang et al. (2006) [31]		*Paeoniae alba *(Bai Shao)	*Citri reticulatae immaturus* (Qin Pi)	*Atractylodes macrocephala* (Bai Zhu)	*Allii macrostemonis* (Xie Bai)	

Leung et al. (2006) [18]		*Atractylodes macrocephala *(Bai Zhu)	*Astragalus membranaceus *(Huang Qi)	*Paeonia lactiflora *(Bai Shao)	*Atractylodes chinensis* (Cang Zhu)	*Bupleurum chinense* (Chai Hu)
		*Citrus reticulata* (Chen Pi)	*Saposhnikovia divaricata *(Fang Feng)	*Murraya paniculata* (Jiu Li Xiang)	*Punica grantum* (Shi Liu Pi)	*Portulaca oleracea* (Ma Chi Xian)
		*Coptis chinensis* (Huang Lian)				

Cheng (2008) [32]		*Paeoniae alba *(Bai Shao)	*Atractylodes macrocephala* (Bai Zhu)	*Saposhnikovia divaricata *(Fang Feng)	And so forth	

Li et al. (2010) [33]		*Paeoniae alba *(Bai Shao)	*Glycyrrhizae preparata *(Zhi Gan Cao)	*Atractylodes macrocephala* (Bai Zhu)	*Citri reticulatae* (Chen Pi)	*Saposhnikovia divaricata *(Fang Feng)
		*Fructus mume *(Wu Mei)				

Chen et al. (2010) [34]		*Paeoniae alba *(Bai Shao)	*Citri reticulatae immaturus* (Qin Pi)	*Atractylodes macrocephala* (Bai Zhu)	*Allii macrostemonis* (Xie Bai)	

Tang et al. (2011) [35]		Astragalus membranaceus (Huang Qi)	*Paeoniae alba *(Bai Shao)	*Atractylodes macrocephala* (Bai Zhu)	*Citri reticulatae *(Chenpi)	*Coptidis* (Huang Lian)
		*Zingiberis preparata *(Paojiang)	*Aucklandiae* (Mu Xiang)	*Saposhnikovia divaricate *(Fang Feng)	*Myristicae *(Rou Doukou)	

Li (2011) [36]		*Semen Myristicae *(Rou Dou Kou)	*Fructus Psoraleae *(Bu Gu Zhi)	*Bructus Schisandrae Chinensis *(Wu Wei Zi)	*Fructus Evodiae Rutaecarpae *(Wu Zhu Yu)	*Radix Codonopsis* (Dang Shen)
		*Rhizoma Atractylodis Macrocephalae* (Bai Zhu)	*Radix Curcumae Wenyujin *(Yu Jin)	*Rhizoma Zinjiberis Recens* (Sheng jiang)	*Fructus Jujubae* (Da Zao)	

Su et al. (2013) [37]		*Semen Myristicae *(Rou Dou Kou)	*Fructus Psoraleae *(Bu Gu Zhi)	*Bructus Schisandrae Chinensis *(Wu Wei Zi)	*Fructus Evodiae Rutaecarpae *(Wu Zhu Yu)	*Radix Codonopsis* (Dang Shen)
		*Rhizoma Atractylodis Macrocephalae* (Bai Zhu)	*Radix Curcumae Wenyujin *(Yu Jin)	*Rhizoma Zinjiberis Recens* (Sheng Jiang)	*Fructus Jujubae* (Da Zao)	

Cai et al. (2012) [38]		*Codonopsis pilosulab * (Dang Shen)	*Paeoniae alba *(Bai Shao)	*Atractylodes macrocephala * (Bai Zhu)	*Poria cocos* (Fu Ling)	* Curcumae wenyujin *(Yu Jin)
		*Glycyrrhizae preparata *(Zhi Gan Cao)	*Alpiniae katsumadai *(Cao Dou Kou)	*Saposhnikovia divaricate *(Fang Feng)	*Lablab album* (Bai Bian Dou)	*Citri reticulatae *(Chen Pi)
		*Amomum villosum* (Sha Ren)	*Albiziae* (He Huan Pi)	*Platycodon grandiflorum *(Jie Geng)	*Semen coicis* (Yi Yi Ren)	

Ko et al. (2013) [39]		*Agastache rugosa *(Huo Xiang)	*Perilla frutescens *(Zhi Su)	*Angelica dahurica *(Bai Zhi)	*Areca catechu *(Bing Lang)	*Poria cocos *(Fu Ling)
		*Pinellia temate *(Ban Xia)	*Magnolia officinalis *(Hou Po)	*Atractylodes macrocephala* (Bai Zhu)	*Citrus unshiu *(Chen Pi)	*Zingiber officinale *(Sheng Jiang)
		*Ziziphus jujube *(Da Zao)	*Platycodon grandiflorum *(Jie Geng)	*Glycyrrhizae uralensis* (Zhi Gan Cao)		

Li et al. (2014) [40]		*Paeoniae alba *(Bai Shao)	*Glycyrrhizae preparata *(Zhi Gan Cao)	*Atractylodes macrocephala* (Bai Zhu)	*Citri reticulatae* (Chen Pi)	*Saposhnikovia divaricata *(Fang Feng)
		*Fructus mume *(Wu Mei)				

**Table 3:** Global symptom improvement, CHM versus placebo.

Therapy	Study	Response rate, % (response/*N*)		Therapeutic gain, %	NNT	RR (95% CI)
		CHM	Placebo			
*Individualized*	Bensoussan et al. 1998 [27]^I^	**47.4 (18/38)**	**29.4 (5/17)**	**18.0**	**5.6**	**1.61 (0.72, 3.62)**
*SLIS*		**65.2 (152/233)**	**46.9 (97/207)**	**18.3**	**5.5**	**1.42 (1.08, 1.86)**
	Bensoussan et al. 1998 [27]^S^	67.4 (29/43)	33.3 (6/18)	34.1	2.9	2.02 (1.02, 4.02)
	Leung et al. 2006 [18]	35.0 (21/60)	44.1 (26/59)	−9.1	—	0.79 (0.51, 1.24)
	Li et al. 2010 [33]	83.3 (25/30)	60.0 (18/30)	23.3	4.3	1.39 (1.00, 1.94)
	Li et al. 2014 [40]	72.5 (58/80)	45.0 (36/80)	27.5	3.6	1.61 (1.22, 2.13)
	Luo 2002 [28]	95.0 (19/20)	55.0 (11/20)	40.0	2.5	1.73 (1.15, 2.60)
*WKIS*		**90.0 (144/160)**	**43.8 (70/160)**	**46.2**	**2.2**	**2.06 (1.71, 2.47)**
	Li 2011 [36]	92.5 (37/40)	42.5 (17/40)	50.0	2	2.18 (1.50, 3.15)
	Su et al. 2013 [37]	89.2 (107/120)	44.2 (53/120)	45.0	2.2	2.02 (1.64, 2.49)

*Total*		**72.9 (314/431)**	**44.8 (172/384)**	**28.1**	**3.6**	**1.62 (1.31, 2.49)**

CHM, Chinese herbal medicine; NNT, number needed to treat; RR, relative risk; SLIS, soothing the liver and invigorating the spleen; WKIS, warming the kidney and invigorating the spleen; I, individualized group; S, standard group.

## References

[1] Chang J. Y., Talley N. J. (2010). Current and emerging therapies in irritable bowel syndrome: from pathophysiology to treatment. *Trends in Pharmacological Sciences*.

[2] Longstreth G. F., Thompson W. G., Chey W. D., Houghton L. A., Mearin F., Spiller R. C. (2006). Functional bowel disorders. *Gastroenterology*.

[3] Lovell R. M., Ford A. C. (2012). Global prevalence of and risk factors for irritable bowel syndrome: a meta-analysis. *Clinical Gastroenterology and Hepatology*.

[4] Rusu F., Dumitrascu D. L. (2015). Epidemiology of irritable bowel syndrome in the former communist countries from Eastern Europe: a systematic review. *Clujul Medical*.

[5] Pan G. Z., Lu S., Ke M. Y., Han S., Guo H., Fang X. C. (2000). An epidemiologic study of irritable bowel syndrome in Beijing—a stratified randomized study by clustering sampling. *Zhonghua Liu Xing Bing Xue Za Zhi*.

[6] Xiong L.-S., Chen M.-H., Chen H.-X., Xu A.-G., Wang W.-A., Hu P.-J. (2004). A population-based epidemiologic study of irritable bowel syndrome in Guangdong province. *Zhonghua Yi Xue Za Zhi*.

[7] Everhart J. E., Ruhl C. E. (2009). Burden of digestive diseases in the United States part I: overall and upper gastrointestinal diseases. *Gastroenterology*.

[8] Soares R. L. S. (2014). Irritable bowel syndrome: a clinical review. *World Journal of Gastroenterology*.

[9] Zijdenbos I. L., Wit N. J., Heijden G. J., Rubin G., Quartero A. O. (2009). Psychological treatments for the management of irritable bowel syndrome. *The Cochrane Database of Systematic Reviews*.

[10] Xie C., Tang Y., Wang Y. (2015). Efficacy and safety of antidepressants for the treatment of irritable bowel syndrome: a meta-analysis. *PLoS ONE*.

[11] Annaházi A., Róka R., Rosztóczy A., Wittmann T. (2014). Role of antispasmodics in the treatment of irritable bowel syndrome. *World Journal of Gastroenterology*.

[12] Trinkley K. E., Nahata M. C. (2011). Treatment of irritable bowel syndrome. *Journal of Clinical Pharmacy and Therapeutics*.

[13] Li C.-Y., Li S.-C. (2015). Treatment of irritable bowel syndrome in China: a review. *World Journal of Gastroenterology*.

[14] Canavan C., West J., Card T. (2015). Change in quality of life for patients with irritable bowel syndrome following referral to a gastroenterologist: a cohort study. *PLoS ONE*.

[15] Saito Y. A., Rey E., Almazar-Elder A. E. (2010). A randomized, double-blind, placebo-controlled trial of St John's wort for treating irritable bowel syndrome. *American Journal of Gastroenterology*.

[16] Chang F.-Y., Lu C.-L. (2009). Treatment of irritable bowel syndrome using complementary and alternative medicine. *Journal of the Chinese Medical Association*.

[17] Ma Y. X., Liu X., Liu C. Z. (2013). Randomized clinical trial: the clinical effects of herb-partitioned moxibustion in patients with diarrhoea-predominant irritable bowel syndrome. *Evidence-Based Complementary and Alternative Medicine*.

[18] Leung W. K., Wu J. C. Y., Liang S. M. (2006). Treatment of diarrhea-predominant irritable bowel syndrome with traditional Chinese herbal medicine: a randomized placebo-controlled trial. *The American Journal of Gastroenterology*.

[19] Brinkhaus B., Hentschel C., Von Keudell C. (2005). Herbal medicine with curcuma and fumitory in the treatment of irritable bowel syndrome: a randomized, placebo-controlled, double-blind clinical trial. *Scandinavian Journal of Gastroenterology*.

[20] Liu J. P., Liu Y. X., Wei M. L., Yang M., Grimsgaard S. (2006). Herbal medicines for treatment of irritable bowel syndrome. *Cochrane Database of Systematic Reviews*.

[21] Shi J., Tong Y., Shen J.-G., Li H.-X. (2008). Effectiveness and safety of herbal medicines in the treatment of irritable bowel syndrome: a systematic review. *World Journal of Gastroenterology*.

[22] Xiao Y., Liu Y., Huang S. (2015). The efficacy of Shugan Jianpi Zhixie therapy for diarrhea-predominant irritable bowel syndrome: a meta-analysis of randomized, double-blind, placebo-controlled trials. *PLoS ONE*.

[23] Li Q., Yang G.-Y., Liu J.-P. (2013). Syndrome differentiation in chinese herbal medicine for irritable bowel syndrome: a literature review of randomized trials. *Evidence-Based Complementary and Alternative Medicine*.

[24] Higgins J. P. T., Green S. (2011). *Cochrane Handbook for Systematic Reviews of Interventions*.

[25] Higgins J. P. T., Thompson S. G., Deeks J. J., Altman D. G. (2003). Measuring inconsistency in meta-analyses. *British Medical Journal*.

[26] DerSimonian R., Laird N. (1986). Meta-analysis in clinical trials. *Controlled Clinical Trials*.

[27] Bensoussan A., Talley N. J., Hing M., Menzies R., Guo A., Ngu M. (1998). Treatment of irritable bowel syndrome with Chinese herbal medicine: a randomized controlled trial. *The Journal of the American Medical Association*.

[28] Luo T. J. (2002). *Treatment of Irritable Bowel Syndrome with Shu Gan Jian Pi Decoction: A Randomized Trial*.

[29] Shen J., Zhu Q., Yuan Z. Y., Zhang Z. W., Chen K. M. (2005). Effect of Chang Ji An oral liquid on stimulated functional area of brain in patients of diarrhea-predominant irritable bowel syndrome of disharmony between liver and spleen. *Chinese Journal of Integrated Traditional and Western Medicine On Gastro-Spleen*.

[30] Shen J., Zhu Q., Yuan Y.-Z., Zhang Z. W., Chen K. M. (2005). Effect of Chang Ji an oral liquid on activated signal alterative intensity in algesthesia domain in patients with diarrhea type irritable bowel syndrome due to Gan-Pi disharmony. *Chinese Journal of Integrated Traditional and Western Medicine*.

[31] Wang G., Li T. Q., Wang L., Xia Q., Cheng Y., Zhang R. M. (2006). Tongxiening granule in the treatment of diarrhea-predominant irritable bowel syndrome(stagnation of the liver-Qi attacking the spleen): a prospective, randomized, placebo-controlled, double-blind clinical trial. *Chinese Journal of Evidence-Based Medicine*.

[32] Cheng H. H. (2008). *The Effects of Soothing Liver and Activating Spleen Method on the Visceral Hypersensitivity of Diarrhea-Predominant Irritable Bowel Syndrome and the Research of this Mechanism*.

[33] Li Y. M., Zhang Y. N., Cai J., Lin J. (2010). A randomized, double-blinded and placebo-controlled trial of ‘Chang Ji Tai Granule’ in treating diarrhea-predominant irritable bowel syndrome. *Shanghai University of Traditional Chinese Medicine*.

[34] Chen D. F., Xia Q., Gong M., Liu X. L., Jiang Y. P. (2010). Effects of Tong Xie Ning granule in treatment of diarrhea-predominant irritable bowel syndrome: a randomized, double-blind, placebo-controlled, multicenter study. *Chinese Journal of Digestion*.

[35] Tang X. D., Li Z. H., Li B. S., Gao R., Wang F. Y., Lu F. Treatment of diarrhea-predominant irritable bowel syndrome with Chang An Yi Hao decoction: a randomized, double-blinded and placebo-controlled trial.

[36] Li Y. F. (2011). *Clinical Observation of Warmming the Kidney and Fortifying the Spleen Therapy in Treating Diarrhea-Predominant Irritable Bowel Syndrome*.

[37] Su X. L., Tang Y. P., Zhang J. (2013). Curative effect of warming kidney and fortifying spleen recipe on diarrhea-predominant irritable bowel syndrome. *Journal of Traditional Chinese Medicine*.

[38] Cai L. J., Lv B., Meng L. N., Ma L. J., Fan Y. H. (2012). Treatment of diarrhea-predominant irritable bowel syndrome with Shu Gan Jian Pi Wen Shen decoction: a randomized controlled trial. *Chinese Archives of Traditional Chinese Medicine*.

[39] Ko S.-J., Han G., Kim S.-K. (2013). Effect of Korean herbal medicine combined with a probiotic mixture on diarrhea-dominant irritable bowel syndrome: a double-blind, randomized, placebo-controlled trial. *Evidence-Based Complementary and Alternative Medicine*.

[40] Li Y. M., Lin J., Cai J., Chen M. X. (2014). A randomized, double-blinded, placebo-controlled research on Chang Ji Tai combined with percutaneous accupoint stimulation in the treatment of diarrhea-predominant irritable bowel syndrome. *Chinese Journal of Integrated Traditional and Western Medicine*.

[41] Xiao H.-T., Zhong L., Tsang S.-W., Lin Z.-S., Bian Z.-X. (2015). Traditional Chinese medicine formulas for irritable bowel syndrome: from ancient wisdoms to scientific understandings. *American Journal of Chinese Medicine*.

[42] Su X. L., Wei R. H., Wei W., Zhang J., Bai Y. B., Shi H. X. (2015). Effect of Shen warming Pi strengthening method on the expression of serum T cell subsets in IBS-D rats. *Chinese Journal of Integrative Medicine*.

[43] Karantanos T., Markoutsaki T., Gazouli M., Anagnou N. P., Karamanolis D. G. (2010). Current insights in to the pathophysiology of irritable bowel syndrome. *Gut Pathogens*.

